# Organizing Pneumonia as First Manifestation of Seronegative Rheumatoid Arthritis

**DOI:** 10.7759/cureus.26679

**Published:** 2022-07-09

**Authors:** Ola Al-Jobory, Job Varghese, Anass Dweik, Mustafa Al-bayati, Nichole Davey

**Affiliations:** 1 Internal Medicine, Texas Tech University Health Sciences Center, Amarillo, USA; 2 Rheumatology, Allergy ARTS (Asthma, Rheumatology Treatment Specialists) Clinic, Amarillo, USA

**Keywords:** connective tissue disease, ctd, interstitial lung disease, ild, rheumatoid arthritis, ra

## Abstract

Rheumatoid arthritis is a multisystem autoimmune inflammatory disease that typically presents as a case of arthritis that can later progress to involve multiple organs. Interstitial lung disease is one of the many extra-articular manifestations of rheumatoid arthritis. We discuss a case of rheumatoid arthritis that presented with interstitial lung disease prior to the development of arthritis along with the discussion of novel anti-carbamylated protein antibody as a serological marker for the diagnosis of seronegative rheumatoid arthritis.

## Introduction

Rheumatoid arthritis (RA) is a multisystem autoimmune inflammatory disease that affects multiple organs, including the lungs, kidneys, joints, and heart. One of the many manifestations of RA in the lungs is the development of interstitial lung disease (ILD) [1]. Despite the fact that RA is more prevalent in females, RA-associated ILD is in fact more common in males, with a male-to-female ratio of 2:1. Typically, lung involvement in RA tends to develop after articular manifestations; however, it can occasionally (i.e., 10-20% of cases) precede articular manifestations [2]. Here, we present a case of RA with an initial manifestation of organizing pneumonia, a rare form of ILD, which was diagnosed with a novel anti-carbamylated protein (anti-CarP) antibody, as the other typical factors (antinuclear antibody (ANA), Scl-70, myositis, rheumatoid factor, and anti-cyclic citrullinated peptide) were negative.

## Case presentation

**Figure 1:**
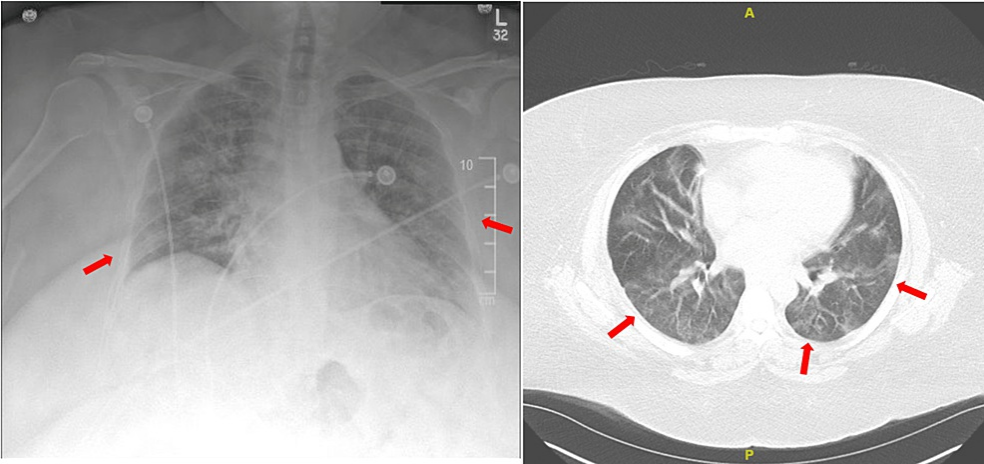
Radiographic imaging including a simple chest x-ray and CT scan showing extensive ground-glass opacities

A 38-year-old obese female with a medical history of childhood asthma presented to the hospital with shortness of breath and a worsening cough. She was admitted to the hospital under different providers multiple times over the last six months with a presumed diagnosis of acute hypoxic respiratory failure (AHRF) secondary to suspected pneumonia. She received steroids and antibiotics and required intubation during some of her admissions. On her current visit, she endorsed being recently discharged from a nearby hospital for another episode of AHRF. On presentation, her vitals were significant for tachypnea with a respiratory rate of 30 breaths/minute and tachycardia with a heart rate of 110 beats/minute. Physical examination revealed acute cardiopulmonary distress with bilateral expiratory wheezing. Her musculoskeletal examination was only significant for mild pain in active and passive movement of her metacarpophalangeal (MCP) joints. Rapid sequence intubation was done immediately to relieve her respiratory distress. Laboratory studies showed no elevation in either leukocyte count or procalcitonin. A chest x-ray revealed bilateral diffuse coarse interstitial opacities. A computed tomography (CT) angiogram was also done, which showed moderate ground glass densities throughout both lungs (Figure 1) with no evidence of pulmonary embolism. A transthoracic echocardiogram was obtained to rule out cardiac etiology, and the patient was reported to have an ejection fraction of 55-60% with mild concentric left ventricular hypertrophy. Pulmonology was consulted, and they were able to find an old pulmonary function test performed two years prior that suggested restrictive lung disease that missed being followed up. Given the overall complexity of the patient’s condition, the decision was made to consult thoracic surgery for a video-assisted thoracoscopic surgery to obtain a biopsy. The procedure was done, and the pathology report revealed evidence of organizing pneumonia. The patient was kept on methylprednisolone (40 mg every six hours) along with a combination nebulization of albuterol-ipratropium (3 mL every six hours) as well. She later improved and was successfully extubated with close monitoring. The patient was discharged and was advised to follow up with a rheumatology appointment. On our follow-up during her appointment, she had an extensive autoimmune workup done, which was negative for ANA, Scl-70, myositis, rheumatoid factor, and anti-cyclic citrullinated peptide (anti-CCP). C3 and C4 levels were within normal limits. Anti-CarP antibody was positive, and she was labeled as a case of seronegative RA with ILD.

## Discussion

The inner lining of the joint capsule, also known as the synovium, is the principal site that is affected by immune and inflammatory responses in RA. Nevertheless, other tissues can also be affected, including the lungs. The clinical spectrum of lung disease is rather non-specific, ranging from mild reversible ILD to rapidly progressive fibrotic conditions that can carry a poor prognosis and a higher mortality rate. The most common manifestation of connective tissue disease in the lungs is usual interstitial pneumonia, which represents approximately 37% of cases. Other less common manifestations include nonspecific interstitial pneumonia, which is seen in 30% of cases, obliterative bronchiolitis in 17% of cases, and organizing pneumonia (OP) in 8% of cases [3]. Our patient had OP, which is thought to develop from autoimmune damage to both the alveolar wall and capillary endothelium lining, resulting in capillary leakage of plasma proteins including coagulation factors with deposition of fibrin on alveolar surfaces. Those changes are often represented as granulation tissue plugs within alveolar walls in microscopy [4].

The diagnosis of RA requires the presence of: (a) inflammatory arthritis in three or more joints, (b) positive serology, including rheumatoid factor, anti-CCP, anti-citrullinated peptide antibody (ACPA), (c) elevated acute phase proteins, including either C-reactive protein and/or erythrocyte sedimentation rate, (d) symptom duration for at least more than six weeks, and (e) exclusion of other similar etiologies that can also present with joint diseases, such as systemic lupus erythematosus and psoriatic arthritis

Typically, the rheumatoid factor serves as the main serological marker, as it carries a sensitivity of 60-90% with a specificity of 85%. Anti-CCP also carries a reasonable sensitivity that ranges between 65% and 85% with a specificity of 98%, and it can also be used as either an adjuvant or in place of RF, especially when RF serology is negative. Despite the high diagnostic value of ACPA and RF, there is a need for additional serological markers to aid in the diagnosis and management of RA [5], especially when those two antibodies are negative and the condition would be labeled as a seronegative RA. It is helpful to check for anti-CarP antibodies, as they have a sensitivity of 44%, but they have a specificity of 89%. CarP antibodies target carbamylated proteins that convert lysine into homocitrulline under the influence of cyanate [6]. The presence of anti-CarP antibody helps detect RA prior to the presence of joint symptoms; thus, it can help in the early detection of RA, and this can affect the prognosis by starting disease-modifying anti-rheumatic drugs (DMARDs) early [7].

Our patient developed extra-articular manifestations of RA prior to articular symptoms. However, it remains unclear whether our patient was having articular symptoms secondary to the steroids that she was receiving during her frequent admissions for AHRF, which were suspected to be secondary to recurrent pneumonia, or whether she simply had a worsening of her articular disease with time, which later led her to complain of MCP joint pain.

## Conclusions

RA is an autoimmune disease that can affect multiple organs and may be non-specific in presentation. It mainly presents with articular symptoms first followed by extra-articular manifestations. However, there are instances where extra-articular manifestations may precede articular symptoms. As a result, the diagnosis of RA may be delayed, leading to a delay in the administration of DMARDs. Clinicians should have a low threshold to order serologic markers in patients with a high index of suspicion for RA. Anti-CarP antibody should be considered as well given the fact that the usual serologies ordered for RA may not necessarily return positive and the condition may remain undiagnosed, leading to a delay in therapy and permanent intra-articular and extra-articular damage.
